# Commentary: Effect of aspirin in patients with established asymptomatic carotid atherosclerosis: a systematic review and meta-analysis

**DOI:** 10.3389/fphar.2023.1142248

**Published:** 2023-06-29

**Authors:** J. David Spence

**Affiliations:** Western University, London, ON, Canada

**Keywords:** aspirin, atherosclerosis, carotid, intima and media thickness, antiplatelet agents, cardiovascular risk

## Introduction

There was a key flaw in the review by Hu et al. (2022), who reported that aspirin did not slow progression of carotid intima-media thickness (IMT) and did not reduce the risk of cardiovascular events in patients with carotid IMT.

## Discussion

The authors studied IMT, which is biologically, (Spence, 2015), genetically, (Pollex and Hegele, 2006), and pathologically (Finn et al., 2010) distinct from atherosclerosis (Spence, 2020). The journal Atherosclerosis has established a policy that IMT must not be referred to as “preclinical atherosclerosis”. It should be referred to as “arterial injury” or “arteriopathy”, not “atherosclerosis” (Raggi and Stein, 2020).

IMT is a much weaker predictor of myocardial infarction (Johnsen et al., 2007) or stroke (Mathiesen et al., 2011) than carotid plaque burden, measured as total plaque area (TPA). Indeed, carotid plaque burden is strongly associated with (Sillesen et al., 2012), and as predictive of cardiovascular risk (Baber et al., 2015) as a coronary calcium score. IMT is neither (Sillesen et al., 2012; Baber et al., 2015).

## Conclusion

Among patients referred for cardiovascular prevention whose TPA was in the top quartile (>119 mm^2^), the 5-year risk of stroke, myocardial infarction or vascular death was 19.5%, after adjusting for baseline risk factors (Spence et al., 2002). It is extremely likely that aspirin would reduce cardiovascular risk in such high-risk patients with true atherosclerosis.
